# Social effects on AVT and CRF systems

**DOI:** 10.1007/s10695-021-00995-w

**Published:** 2021-09-02

**Authors:** Tobias Backström, Per-Ove Thörnqvist, Svante Winberg

**Affiliations:** 1 Institute of Integrated Natural Sciences, University Koblenz-Landau, Koblenz, Universitätsstraße 1, 56070 Koblenz, Germany; 2 Behavioural Neuroendocrinology Lab, Department of Neuroscience, Biomedical Centre (BMC), Uppsala University, Box 572, SE-751 23 Uppsala, Sweden

**Keywords:** Aggression, Arginine-vasotocin (AVT), Corticotropin-releasing factor (CRF), AVT receptor, CRF receptor, Social stress

## Abstract

Stress and aggression have negative effects on fish welfare and productivity in aquaculture. Thus, research to understand aggression and stress in farmed fish is required. The neuropeptides arginine-vasotocin (AVT) and corticotropin-releasing factor (CRF) are involved in the control of stress and aggression. Therefore, we investigated the effect of agonistic interactions on the gene expression of AVT, CRF and their receptors in juvenile rainbow trout (*Oncorhynchus mykiss*). The social interactions lead to a clear dominant-subordinate relationship with dominant fish feeding more and being more aggressive. Subordinate fish had an upregulation of the AVT receptor (AVT-R), an upregulation of CRF mRNA levels, and higher plasma cortisol levels. The attenuating effect of AVT on aggression in rainbow trout is proposed to be mediated by AVT-R, and the attenuating effect of the CRF system is proposed to be mediated by CRF.

## Introduction

Stress, and stress-related behaviour, is a problem in aquaculture as it results in lowered productivity and reduced welfare of farmed fish (The EFSA Journal 2009). The general stress response of teleost fish is well studied (Wendelaar Bonga 1997), and in aquaculture the stress is often related to aggressive behaviour (Bergqvist and Gunnarsson 2011). Thus, research concerning aggression and understanding processes involved in aggression is necessary. In recent years, the neuropeptides arginine-vasopressin (AVP) in mammals, or the non-mammalian homologue arginine-vasotocin (AVT), and corticotropin-releasing factor (CRF) have been suggested as factors modulating agonistic behaviour and aggression in vertebrates, including teleost fish.

The AVP/AVT is involved in the stress response as an adrenocorticotropin (ACTH) secretagogue in the hypothalamic–pituitary–adrenal (HPA) axis in mammals, and in the hypothalamic-pituitary-interrenal (HPI) axis in teleost fish (Fryer et al. 1985; Gillies et al. 1982; Tonon et al. 1986). Moreover, several behaviours, including aggressive behaviour, seem to be modulated by the AVP/AVT system [see reviews by Balment et al. (2006), Godwin and Thompson (2012), and Goodson and Bass (2001)]. The effects of AVP are mediated by the V_1A_, V_1B_ and V_2_ receptors (Balment et al. 2006; Godwin and Thompson 2012; Manning et al. 2008), and recently the homologues of these receptors have been cloned in teleost fish (Konno et al. 2010; Lema 2010). The V_1A_ receptor and its non-mammalian homologue, the AVT receptor (AVT-R), are involved in the behavioural responses to stress (Castagna et al. 1998; Goodson and Bass 2001).

Corticotropin-releasing factor is also involved in the stress response as an ACTH secretagogue in the HPA/HPI axis (Gillies et al. 1982; Vale et al. 1981). Furthermore, the CRF system is an important mediator of several behavioural stress responses (see reviews by Bale and Vale (2004), Heinrichs and Koob (2004), Lowry and Moore (2006)), as well as aggression in vertebrates [see review by Backström and Winberg (2013)]. The effects of CRF are mediated by at least two different receptors, the CRF receptor 1 (CRF-R1) and the CRF receptor 2 (CRF-R2) [see reviews by Smagin et al. (2001) and Heinrichs and Koob (2004)].

Based on this background, we performed a study to evaluate the effects of aggression on AVT and CRF systems. To study the effects of social dominance on the mRNA expression of the AVT and CRF systems in teleost fish, we used juvenile rainbow trout *Oncorhynchus mykiss* (Walbaum, 1792) which are territorial and form strong dominance hierarchies (Chiszar et al. 1975; McIntyre et al. 1979). Previous studies have reported that aggression is attenuated by exogenous AVT (Backström and Winberg 2009) and CRF (Backström et al. 2011a) in rainbow trout. Similarly, mRNA levels of CRF have been reported to be upregulated in subordinate individuals (Bernier et al. 2008; Doyon et al. 2003). However, the effects of aggressive behaviour and dominance relationships upon mRNA expressions of AVT, AVT-R, CRF-R1 and CRF-R2 at the same time have not been examined in this species. Based on the studies using exogenous AVT, we hypothesized that mRNA expression of AVT and/or AVT-R would be upregulated in subordinate individuals. Similarly, our hypothesis concerning the CRF system was an upregulation of mRNA expression of CRF, CRF-R1 and/or CRF-R2 in subordinates based on the exogenous effect of CRF.

Here, we report the effects of 5 days inter-individual interactions in quadruples, thus establishing dominance–subordinance relationship, upon mRNA expression of AVT, CRF and their respective receptors. Furthermore, aggressive and foraging behaviours during the 5 days of interactions were monitored, and plasma cortisol levels were quantified to evaluate stress levels.

## Materials and methods

### Experimental animals

The experiments were performed on juvenile rainbow trout weighing 83.0 ± 15.2 g (mean ± SD, *N* = 27). Prior to the experiment, fish were kept indoors in a 1-m^3^ holding tank at the Evolutionary Biology Centre, Uppsala University, at a rearing density of approximately 0.02 kg/L for at least 1 week prior to the experiment. The holding tank was continuously supplied with aerated (> 90% O_2_ saturation) Uppsala tap water (pH 7.6, HCO_3_^−^ 5.2 mM, Ca^2+^ 2.8 mM, Mg^2+^ 0.4 mM) at 8–11 °C and the light/dark regime was continuously and automatically adjusted to latitude 51°N conditions. Fish were hand-fed with commercial trout pellets (EWOS ST40, Ewos AS, Bergen, Norway) at 1–2% of body mass per day.

### Experimental protocol—dominance hierarchy

Juvenile rainbow trout randomly selected from the holding tank were lightly anaesthetized with ethyl-4-amino benzoate (0.25 g/L), weight matched in quadruples (deviation in weight less than 15%), tagged by either no, dorsal, ventral or dorsal and ventral cut in the caudal fin. Fish were socially isolated for 1 week in individual compartments, created by removable dark PVC walls in experimental aquaria (250 L), and continuously supplied with aerated Uppsala tap water (oxygen saturation > 90%, 0.8 L/min, 8–11 °C). Individual compartments were equally sized at 62.5 L (250 × 500 × 500 mm). The light/dark regimen was 12-h light/12-h dark (light on at 08.00 and light off at 20.00 h), light provided by a 30-W Lumilux daylight fluorescent tube placed 40 cm above the water surface of each aquarium. During social isolation, fish were fed commercial trout pellets (EWOS ST40) corresponding to 1% of their body mass per day. At the end of isolation, all individuals ate the entire ration.

Following isolation, the PVC walls were removed and a total of 8 quadruples were allowed to interact. Also, a PVC tube (length 25–27 cm, outer diameter 7.6 cm, inner diameter 6.7 cm, weighted down with a 50-mL Falcon tube filled with gravel) was put in each aquarium with the purpose to act as a hiding place. Interaction was continuous over 5 days and fish were fed the sum of earlier rations at a specific place of the aquaria. Agonistic interactions were observed in all quadruples for three different 5-min periods per day, the first in the morning 10.30–11.10, the second in the afternoon prior to feeding 15.10–15.50 and the third in the afternoon post feeding 15.50–16.30. During each 5-min period, the number of attacks performed per individual, as well as individuals receiving these attacks, was counted, and the number of times spent in or in the vicinity of the hiding place for every fish and an estimation of social rank based on general behaviour and demeanour were noted. The estimation of rank was mainly based on attacks performed; thus, the one performing most attacks in one aquarium was the alpha, the one with the second most attacks was the beta, and so on for gamma and delta. Furthermore, attacks received were also used to distinguish when two individuals had a similar number of performed attacks. Five individual fish were too badly injured by the interactions, and were removed from the aquarium and further analysis. During feeding, every individual eating got a score of 1 per day leading to a maximum possible score of 5. After 5 days, the fish were netted in estimated ranking order (alpha, beta, gamma and delta) and sacrificed using ethyl-4-amino benzoate (0.5 g/L). The fish were weighed and blood collected through the caudal vasculature with a heparinized syringe. Thereafter, the spinal cord was cut, the brain collected and sex was noted. The blood was spun at 16,000 g for 10 min at + 4 °C and the plasma collected, and the brain was wrapped in aluminium foil and frozen on liquid nitrogen. All samples were stored at − 80 °C until analysis.

### Physiological analysis

Plasma was analysed for cortisol using a commercial enzyme linked immunosorbent assay (ELISA) kit (product # 402,710, Neogen Corporation, Lexington, USA, delivered by Skafte Medlab, Onsala, Sweden) as described previously (Backström and Winberg 2009). In short, plasma samples were extracted in ethyl acetate and the organic phase evaporated and dissolved in buffer provided in the kit. The plates included in the kit were exposed to the samples and standards, the enzyme-conjugate and substrate. Prior to reading, 1 N HCl was added to stop enzyme reaction. Absorbance was read at 450 nm. All the samples were assayed in duplicates in one batch and the detection limit was 0.4 ng cortisol/mL, and an intra-assay coefficient of variation of 1–2.18%.

Quantitative PCR (qPCR) was used for analysing mRNA expression as described previously (Backström et al. 2011b). In short, RNA was extracted from brain samples using QIAzol Reagent (QIAGEN), DNAse-treated (DNA-free, Ambion, Austin, USA). The resulting RNA was checked for quality and cDNA was prepared using Stratascript (Stratagene, San Diego, USA). Primers for AVT, AVT-R, CRF, CRF-R1, CRF-R2 and elongation factor 1α (EF1α) as reference gene were used in the qPCR. The primers were 18–22 nucleotides long, melting point around 60 °C and products of 70–110 bp (see Table 1). The primer specificity was tested and efficiencies for primers were 80–100%.

**Table 1:** Primer design for quantitative PCR

Transcript	Accession no. (NCBI)	Forward primer	Reverse primer	Product size, bp		Annealing temp		Efficiency	
CRF	AF296672	ccgatgatccgccgatat	tgttcagcactggacatctc^+^	76		64.64		93.1%	
CRF receptor 1	AJ277157^*^	tcacacccagcaatgtc	gcagtgctctttggccagc	82		62.70		81.3%	
CRF receptor 2	AJ277158^*^	ccaagttgagagcttctacc	aacagcatgtaggtgatccc	103		62.64		92.5%	
Elongation factor 1α	AF498320	gcaggaaaagaacccaacg	agttaccagcagctttcttcc	134		64.65		99.9%	
Vasotocin	X17327^*^	tgaacacacccagaatagagc	tctacttctgctgtgtgtctg	94		64.64		94.4%	
Vasotocin receptor	DQ291141	gtgtagtctggtcctcagc	agtgatgtacgcctttacgc	127		65.65		97.5%	

^*^Primers designed from *O. keta* genes

^+^The sequence for the reverse primer of CRF was as presented here when it was designed, but is now tgttcagcTctggacatctc

### Statistical analysis

All data were tested for normality using Shapiro–Wilk normality test, and if possible transformed to fit normality. Following this process, data was tested using Kruskal–Wallis test (non-parametric data), Welch two-sample one-tailed or two-tailed *t*-test (parametric data) (see “Results” section for motivation of not using the four ranks further). The free software R for statistical computing (R Core Team 2020) in the integrated development environment RStudio (RStudio Team 2019) was used for all statistical analyses. Fin clips (tagging) (Kruskal–Wallis test, *P* = 0.9118), sex (Kruskal–Wallis test, *P* = 0.2892) and weight before (two-tailed *t*-test, *P* = 0.6101) or after (two-tailed *t*-test, *P* = 0.7629) the experiment had no effects on social rank. Data are presented as mean ± s.e.m. if not stated otherwise. The methodology of this study was approved by the Uppsala Animal Research Ethical Committee.

## Results

### Dominance hierarchy

Generally, interactions started directly after removing compartment walls with all four fish involved in performing and receiving attacks. Already at the second 5-min period during the first day of interactions, dominance started to be stabilized with one easily identified alpha individual. However, beta, gamma and delta individuals were not so easily distinguished. When dominance was stabilized, the alpha individual was never usurped. During the study, it was noted that the hiding place was not commonly used, and therefore it was excluded from further analysis. Behavioural analysis of social interactions showed several differences between social ranks (Table 2). Dominant individuals had a higher feeding rate (Kruskal–Wallis test, *P* < 0.001), performed more attacks in the morning (Kruskal–Wallis test, *P* < 0.001) and performed more attacks in the afternoon prior to (Kruskal–Wallis test, *P* < 0.001) and post (Kruskal–Wallis test, *P* < 0.001) feeding. Dominant individuals also received fewer attacks in the morning (two-tailed *t*-test, *P* = 0.004), in the afternoon prior to (Kruskal–Wallis test, *P* < 0.001) and post feeding (Kruskal–Wallis test, *P* = 0.026). These differences were however only between alpha and the other ranks combined with no internal differences between beta, gamma and delta individuals. Therefore, these and the following analyses are divided into dominants, being alpha individuals, and subordinates, i.e. the beta, gamma and delta individuals taken together.

**Table 2:** The total number of attacks performed and received at three different 5-min periods and the total days of feeding during 5 days of social interacting in juvenile rainbow trout

Social rank	Attacks performed during morning	Attacks received during morning	Attacks performed during afternoon prior to feeding	Attacks received during afternoon prior to feeding	Attacks performed during afternoon post feeding	Attacks received during afternoon post feeding	Feeding rate (days/5 days of social interacting)	*N*
Dominant (alpha)	54.1 ± 8.1 *	14.5 ± 2.2	39.6 ± 4.3*	0*	35.9 ± 10.8*	3.0 ± 1.8*	4.125 ± 0.479*	8
Subordinate (beta, gamma and delta combined)	14.7 ± 3.4	26.3 ± 3.1	1.4 ± 1.1	12.3 ± 2.4	2.7 ± 1.6	8.5 ± 2.3	0.816 ± 0.318	19
Beta	16.2 ± 4.0	30.2 ± 6.3	0	10.2 ± 2.2	0	5.3 ± 2.4	0.083 ± 0.083	6
Gamma	16.0 ± 7.5	22.9 ± 5.1	0.9 ± 0.6	15.4 ± 3.1	3.6 ± 3.6	10.9 ± 6.0	1.429 ± 0.759	7
Delta	11.8 ± 6.0	26.5 ± 5.1	3.3 ± 3.3	10.7 ± 6.6	4.3 ± 2.8	8.8 ± 1.7	0.833 ± 0.380	6

Social status is presented in falling order from alpha, beta, gamma to delta. No differences were evident between beta, gamma and delta individuals and they were therefore pooled into one group of subordinates and alpha individuals were termed dominants

Values are mean ± SEM

*Difference between dominant and subordinate ranks (*P* < 0.05, Kruskal–Wallis test or two-tailed *t*-test)

**Fig. 1:**
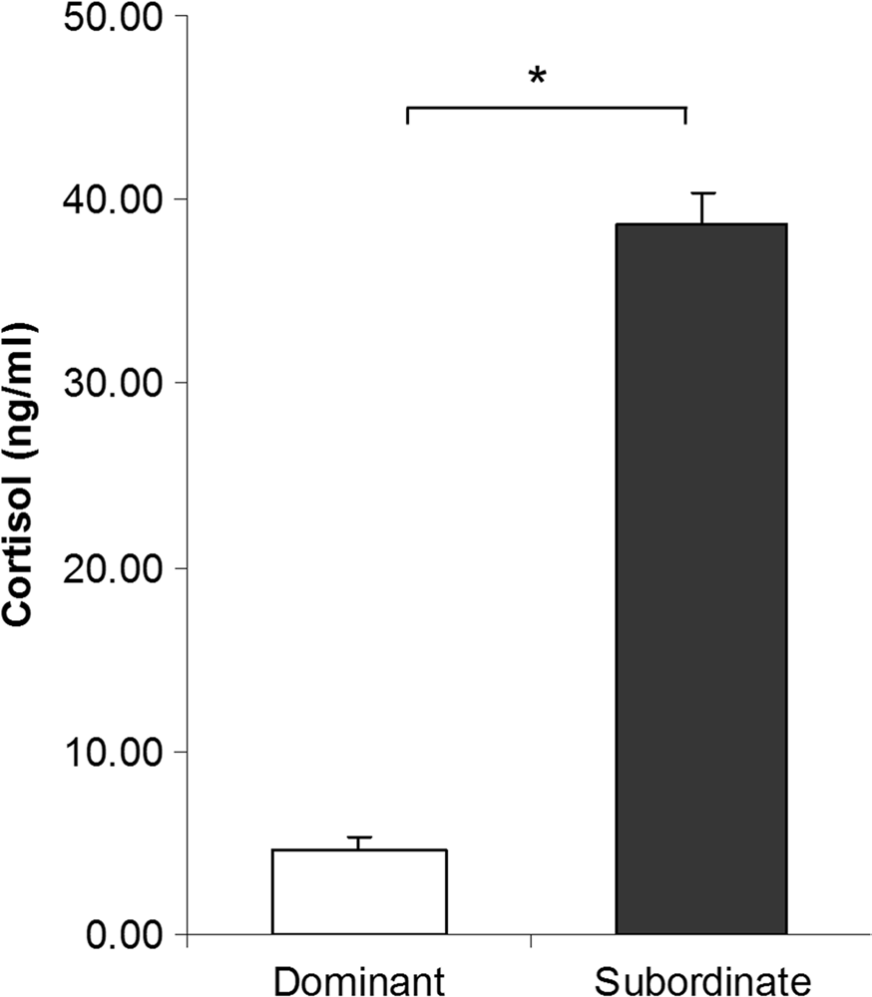
The effect of social status on plasma cortisol levels (ng/ml). Values are mean ± S.E.M and * indicates difference between dominant (*N* = 8) and subordinate individuals (*N* = 19) (*P* < 0.05, Kruskal–Wallis test)

**Fig. 2:**
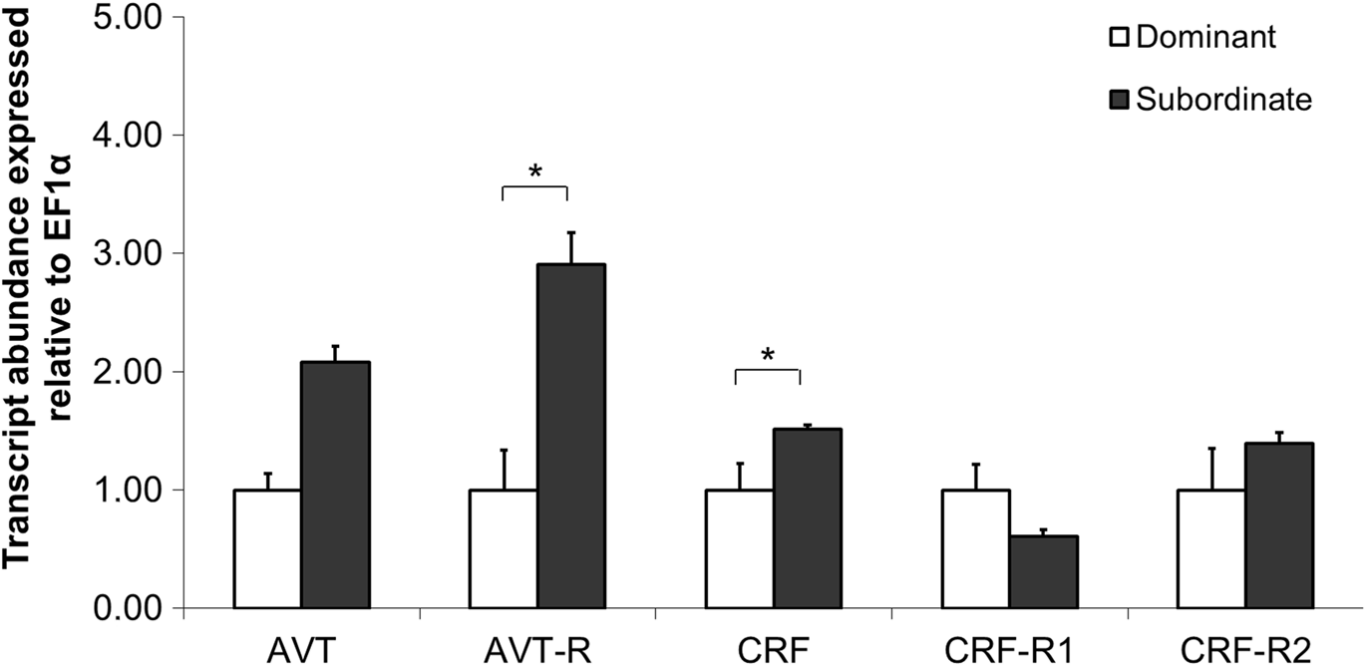
The effect of social status on relative concentrations of AVT, AVT-R, CRF, CRF-R1 and CRF-R2 mRNA (arbitrary values). Values are mean ± S.E.M and * indicates difference between dominant and subordinate individuals (*P* < 0.05, Welch one-tailed *t*-test)

After 5 days of social interaction, plasma cortisol levels were higher in subordinate compared to dominant individuals (Kruskal–Wallis test, *P* = 0.003, Fig. 1). Furthermore, mRNA levels of AVT-R (one-tailed *t*-test, *P* = 0.018, Fig. 2) and CRF (one-tailed *t*-test, *P* = 0.033, Fig. 2) were higher in subordinate compared to dominant individuals. However, there were no significant differences between social ranks in CRF-R1 (one-tailed *t*-test, *P* = 0.929, Fig. 2), CRF-R2 (one-tailed *t*-test, *P* = 0.293, Fig. 2) or AVT (one-tailed *t*-test, *P* = 0.261, Fig. 2) brain mRNA levels.

## Discussion

### Dominance behaviour and the stress axis

A clear dominance hierarchy was established in all groups of fish. Similar to several earlier studies, dominants showed higher food intake and were more aggressive than subordinates [see reviews by da Silva et al. (2021), Fulenwider et al. (2021), Goymann and Wingfield (2004), Sloman and Armstrong (2002), Winberg and Nilsson (1993)]. Furthermore, social subordination induced the typical plasma glucocorticoid elevation seen in previous studies (see reviews by Blanchard et al. (2001), da Silva et al. (2021), Sloman and Armstrong (2002)), and Summers and Winberg (2006)).

### Arginine vasopressin/vasotocin and social behaviour

Arginine vasopressin and the non-mammalian homologue AVT affect social behaviour in vertebrates (Goodson and Bass 2001). Aggression seems to be modulated differently by AVP/AVT depending upon social system in all vertebrates. For instance, AVT stimulates aggression in colonial species (Goodson and Adkins-Regan 1999) and inhibits aggression in territorial species (Goodson 1998) of birds. Similarly, AVP shows divergent effects on aggressive pattern depending on social system in voles of the genera *Microtus* (Young et al. 1997). There is also evidence for diverging AVT effects depending on individual social status. In teleost fish for instance, AVT increases aggressive behaviour in non-territorial bluehead wrasse (*Thalassoma bifasciatum*) males (Semsar et al. 2001) and decreases aggressive behaviour in territorial Amargosa River pupfish (*Cyprinodon nevadensis amargosae*) males (Lema and Nevitt 2004a). Thus, the AVP/AVT effects on aggressive behaviour seem to be evolutionary conserved within specific social systems.

Earlier, we reported an attenuation of aggression by exogenous AVT (Backström and Winberg 2009). Therefore, we predicted an upregulation of AVT expression in brain of subordinate individuals, but no difference was observed between dominants and subordinates in this study. The reason for these non-conclusive results could depend on the use of whole brain for analyses. Several earlier studies show differences in AVP/AVT neurons of specific areas in the brain. AVP/AVT neurons are typically found in the magnocellular and parvocellular cells of the preoptic area*-*anterior hypothalamus (POA-AH) in vertebrates (Goodson and Bass 2001). In mammals, there is evidence that variation in AVP expression in the POA-AH relates to aggressive behaviour. For instance, experimentally induced changes in AVP seem to affect aggression in Syrian hamsters (*Mesocritus auratus*) (Ferris and Delville 1994; Ferris et al. 1986; Ferris and Potegal 1988) and in male prairie voles (*Microtus ochrogaster*) (Gobrogge et al. 2009). Furthermore, AVP distribution differs in the bed nucleus of the stria terminalis (BNST) between two closely related species of the mouse genera *Peromyscus* with divergent aggressive behaviour (Bester-Meredith et al. 1999), and in rats (*Rattus norvegicus*) AVP release in the *paraventricular nucleus* (PVN) differed between active and passive intruders (Ebner et al. 2005). Similar patterns of association between hypothalamic AVT and aggression are also apparent in teleost fish. For instance, in zebrafish (*Danio rerio*), dominant individuals exhibit AVT-immunoreactive cells in magnocellular POA, whereas subordinate individuals exhibit AVT-immunoreactive cells in parvocellular POA (Larson et al. 2006). Furthermore, the territorial multiband butterflyfish (*Chaetodon multicintus*) has larger AVT immunoreactive somata in the POA and higher AVT fibre densities in parts of the telencephalon than the non-territorial milletseed butterflyfish (*Chaetodon miliaris*) (Dewan et al. 2008) and similar findings have been reported concerning different populations with diverging aggressiveness of the Death Valley pupfish (*Cyprinodon nevadensis*) (Lema and Nevitt 2004b). Similarly, in an African cichlid species (*Astatotilapia burtoni*), territorial males exhibit higher levels of AVT mRNA expression in the posterior POA (gigantocellular nucleus) than non-territorial males whereas in the anterior POA (parvocellular nucleus) AVT mRNA expression is lower in territorial males than that in non-territorial males (Greenwood et al. 2008). The AVT distribution is well studied in rainbow trout and the POA is dense with AVT neurons (Saito et al. 2004). Thus, even though our results did not establish a divergence in AVT mRNA expression between dominant and subordinate individuals, these earlier reports indicate that AVT expression could be affected in specific areas of the brain. The expression in these areas, specifically the parvocellular and magnocellular POA, could cancel each other out and therefore not be measurable in whole-brain samples.

In mammals, the behavioural effects of AVP, including aggression, across both social system and social status, seem to be mediated by the V_1A_ receptor. For instance, in *Microtus* voles with different social systems, the brain distribution of V_1A_ receptor also differs and apparently gives this divergence in social system (Young et al. 1997), and male mice (*Mus musculus*) transfected with the *Microtus* vole V_1A_ receptor display similar behavioural effects (Young et al. 1999). In male Syrian hamsters, V_1A_ receptor was upregulated in dominants (Cooper et al. 2005). Similarly, studies using the V_1A_ receptor antagonist Manning compound in teleost fish report behavioural effects. For example, Manning compound decreases courtship and territorial defence in territorial males of bluehead wrasse (*Thalassoma bifasciatum*) (Semsar et al. 2001), and decreases aggression in male beaugregory damselfish (*Stegastes leucostictus*) (Santangelo and Bass 2006). Some indications of Manning compound affecting behaviour in rainbow trout have also been reported. Fish receiving Manning compound prolonged time for dyadic fights when becoming subordinate (Backström and Winberg 2009). Thus, our results that social stress upregulates transcript levels of AVT-R in subordinates fit well with the hypothesis that aggression in territorial animals is attenuated by AVP/AVT.

### Corticotropin-releasing factor, and stress related behaviour

Similar to AVP/AVT, CRF is involved in several behavioural stress responses (Lowry and Moore 2006; Smagin et al. 2001). Among the behaviours affected during stress are feeding and appetite (Bernier 2006; De Pedro et al. 1993; Koob and Heinrichs 1999), aggressive behaviour (Elkabir et al. 1990; Mele et al. 1987) and anxiety-related behaviour (Lowry and Moore 2006; Risbrough and Stein 2006). Furthermore, these responses are usually associated with specific brain areas. For instance, several stressors induce CRF expression in the hypothalamus (Imaki et al. 1991) and PVN of rats (Aubry et al. 1999; Harbuz et al. 1993; Imaki et al. 1991). These patterns are also apparent in teleost fish. For instance, hyperosmotic stress induces CRF expression 24 h post stress in the hypothalamus and the POA of rainbow trout (Craig et al. 2005). Furthermore, repeated chasing stress, isolation stress for at least 24 h and confinement stress for at least 4 h elevated CRF expression in the POA of rainbow trout (Doyon et al. 2005). Interestingly, CRF expression in the POA of rainbow trout was elevated after subordination stress following 72 h of interactions in dyads (Doyon et al. 2003), whereas in *Astatotilapia burtoni* territorial males have higher CRF expression in brain compared to non-territorial males after 4 weeks of social interaction (Chen and Fernald 2008). In our study, CRF was upregulated in subordinate compared to dominant individuals, following the earlier studies as well as our hypothesis.

Aggressive behaviour has been reported to be affected by CRF. For instance, CRF reduced aggression in mice (Mele et al. 1987), whereas low doses of CRF increase aggression and high doses of CRF reduce aggression in rats (Elkabir et al. 1990). In mice, maternal aggression is also reduced by CRF (Gammie et al. 2005) and the two CRF-related peptides Urocortin 1 and Urocortin 3 (D'Anna et al. 2005). Furthermore, the use of CRF antagonists modulates aggressive behaviour. For instance, the CRF-R1 antagonist SSR125543A delayed latency to attack intruders in Syrian hamsters (Farrokhi et al. 2004) and the CRF-R1 antagonist antalarmin reduces defensive posture in socially defeated mice (Robison et al. 2004). In mice selected for aggression, CRF mRNA expression was higher in the less aggressive line 24 h post swim stress (Veenema et al. 2003). Thus, CRF seems to be involved in aggressive behavioural modulation in mammals but not in a consistent pattern. However, the higher CRF expression in subordinates in this study together with an earlier report concerning CRF attenuating aggression (Backström et al. 2011a) supports the hypothesis, although questioned (Carpenter et al. 2009) that CRF reduces aggression and dominance behaviour in rainbow trout.

Corticotropin-releasing factor has at least two different receptors mediating its effects, namely CRF-R1 and CRF-R2 (Bale and Vale 2004; Flik et al. 2006). CRF-R1 is probably involved in the HPA/HPI axis activity and is found in the pituitary gland in teleost fish (Flik et al. 2006). Several studies report that CRF-R1 is involved in stress responses. For instance, CRF-R1-deficient mice have depleted stress response (Bale et al. 2002). In crucian carp (*Carassius carassius*), intraperitoneal injection of antalarmin reduces plasma cortisol during skin extract exposure (Lastein et al. 2008). Huising et al. (2004) reported that CRF-R1 is expressed in the hypothalamus and the pituitary gland, and also showed that CRF-R1 is downregulated after 24 h of restraint stress in the pituitary gland of common carp (*Cyprinius carpio*). Since CRF-R1 seems to be tightly linked to plasma cortisol, differences in expression could be apparent between social ranks. However, in our study, no differences were seen in expression of CRF-R1 mRNA.

On the other hand, CRF-R2 has been connected to the behavioural responses of stress (Flik et al. 2006). Among these behaviours are anxiety-related behaviours, although not exclusively linked to CRF-R2 (Rotzinger et al. 2010). Recently, an anxiety-like behaviour evoked by CRF has been reported in rainbow trout (Carpenter et al. 2007). We also observed a similar head-shake behaviour after administration of both CRF and UI and suggested that the head-shake was mediated by the CRF-R2 (Backström et al. 2011a). In the present study, CRF-R2 expression was not different between dominant and subordinate individuals.

## Conclusions

In conclusion, dominance/subordinance relationship affects AVT-R and CRF expression as well as plasma cortisol. Thus, the apparent attenuating effect on aggression by AVT could be mediated by AVT-R. Furthermore, the attenuating effect of aggression by the CRF system could be mediated by CRF. Finally, more studies are needed to elucidate mRNA expression of AVT, CRF and their respective receptors in specific areas of the brain, and the most promising area seems to be the POA.

## Data Availability

All data are presented in the article.

## References

[CR1] Aubry JM, Bartanusz V, Jezova D, Belin D, Kiss JZ (1999). Single stress induces long-lasting elevations in vasopressin mRNA levels in CRF hypophysiotrophic neurones, but repeated stress is required to modify AVP immunoreactivity. J Neuroendocrinol.

[CR2] Backström T, Winberg S (2009). Arginine–vasotocin influence on aggressive behavior and dominance in rainbow trout. Physiol Behav.

[CR3] Backström T, Winberg S (2013). Central corticotropin releasing factor and social stress. Front Neurosci.

[CR4] Backström T, Pettersson A, Johansson V, Winberg S (2011). CRF and urotensin I effects on aggression and anxiety-like behavior in rainbow trout. J Exp Biol.

[CR5] Backström T, Schjolden J, Øverli Ø, Thörnqvist PO, Winberg S (2011). Stress effects on AVT and CRF systems in two strains of rainbow trout (*Oncorhynchus mykiss*) divergent in stress responsiveness. Horm Behav.

[CR6] Bale TL, Vale WW (2004). CRF and CRF receptors: role in stress responsivity and other behaviors. Annu Rev Pharmacol Toxicol.

[CR7] Bale TL, Picetti R, Contarino A, Koob GF, Vale WW, Lee K-F (2002). Mice deficient for both corticotropin-releasing factor receptor 1 (CRFR1) and CRFR2 have an impaired stress response and display sexually dichotomous anxiety-like behavior. J Neurosci.

[CR8] Balment RJ, Lu W, Weybourne E, Warne JM (2006). Arginine vasotocin a key hormone in fish physiology and behaviour: a review with insights from mammalian models. Gen Comp Endocrinol.

[CR9] Bergqvist J, Gunnarsson S (2011). Finfish aquaculture: animal welfare, the environment, and ethical implications. J Agric Environ Ethics.

[CR10] Bernier NJ (2006). The corticotropin-releasing factor system as a mediator of the appetite-suppressing effects of stress in fish. Gen Comp Endocrinol.

[CR11] Bernier NJ, Alderman SL, Bristow EN (2008). Heads or tails? Stressor-specific expression of corticotropin-releasing factor and urotensin i in the preoptic area and caudal neurosecretory system of rainbow trout. J Endocrinol.

[CR12] Bester-Meredith JK, Young LJ, Marler CA (1999). Species differences in paternal behavior and aggression in peromyscus and their associations with vasopressin immunoreactivity and receptors. Horm Behav.

[CR13] Blanchard RJ, McKittrick CR, Blanchard DC (2001). Animal models of social stress: effects on behavior and brain neurochemical systems. Physiol Behav.

[CR14] Carpenter RE, Watt MJ, Forster GL, Øverli Ø, Bockholt C, Renner KJ, Summers CH (2007). Corticotropin releasing factor induces anxiogenic locomotion in trout and alters serotonergic and dopaminergic activity. Horm Behav.

[CR15] Carpenter RE, Korzan WJ, Bockholt C, Watt MJ, Forster GL, Renner KJ, Summers CH (2009). Corticotropin releasing factor influences aggression and monoamines: modulation of attacks and retreats. Neuroscience.

[CR16] Castagna C, Absil P, Foidart A, Balthazart J (1998). Systemic and intracerebroventricular injections of vasotocin inhibit appetitive and consummatory components of male sexual behavior in Japanese quail. Behav Neurosci.

[CR17] Chen C-C, Fernald RD (2008). Sequences, expression patterns and regulation of the corticotropin-releasing factor system in a teleost. Gen Comp Endocrinol.

[CR18] Chiszar D, Drake RW, Windell JT (1975). Aggressive behavior in rainbow trout (*Salmo gairdneri* Richardson) of two ages. Behav Biol.

[CR19] Cooper MA, Karom M, Huhman KL, Elliott Albers H (2005). Repeated agonistic encounters in hamsters modulate AVP V1a receptor binding. Horm Behav.

[CR20] Craig PM, Al-Timimi H, Bernier NJ (2005). Differential increase in forebrain and caudal neurosecretory system corticotropin-releasing factor and urotensin I gene expression associated with seawater transfer in rainbow trout. Endocrinology.

[CR21] da Silva MC, Canário AVM, Hubbard PC, Gonçalves DMF (2021). Physiology, endocrinology and chemical communication in aggressive behaviour of fishes. J Fish Biol.

[CR22] D'Anna KL, Stevenson SA, Gammie SC (2005). Urocortin 1 and 3 impair maternal defense behavior in mice. Behav Neurosci.

[CR23] De Pedro N, Alonso-Gomez AL, Gancedo B, Delgado MJ, Alonso-Bedate M (1993). Role of corticotropin-releasing factor (CRF) as a food intake regulator in goldfish. Physiol Behav.

[CR24] Dewan AK, Maruska KP, Tricas TC (2008). Arginine vasotocin neuronal phenotypes among congeneric territorial and shoaling reef butterflyfishes: species, sex and reproductive season comparisons. J Neuroendocrinol.

[CR25] Doyon C, Gilmour KM, Trudeau VL, Moon TW (2003). Corticotropin-releasing factor and neuropeptide Y mRNA levels are elevated in the preoptic area of socially subordinate rainbow trout. Gen Comp Endocrinol.

[CR26] Doyon C, Trudeau VL, Moon TW (2005). Stress elevates corticotropin-releasing factor (CRF) and CRF-binding protein mRNA levels in rainbow trout (*Oncorhynchus mykiss*). J Endocrinol.

[CR27] Ebner K, Wotjak CT, Landgraf R, Engelmann M (2005). Neuroendocrine and behavioral response to social confrontation: residents versus intruders, active versus passive coping styles. Horm Behav.

[CR28] Elkabir DR, Wyatt ME, Vellucci SV, Herbert J (1990). The effects of separate or combined infusions of corticotrophin-releasing factor and vasopressin either intraventricularly or into the amygdala on aggressive and investigative behaviour in the rat. Regul Pept.

[CR29] Farrokhi C, Blanchard DC, Griebel G, Yang M, Gonzales C, Markham C, Blanchard RJ (2004). Effects of the CRF1 antagonist SSR125543A on aggressive behaviors in hamsters. Pharmacol Biochem Behav.

[CR30] Ferris CF, Delville Y (1994). Vasopressin and serotonin interactions in the control of agonistic behavior. Psychoneuroendocrinology.

[CR31] Ferris CF, Potegal M (1988). Vasopressin receptor blockade in the anterior hypothalamus suppresses aggression in hamsters. Physiol Behav.

[CR32] Ferris CF, Meenan DM, Axelson JF, Albers HE (1986). A vasopressin antagonist can reverse dominant/subordinate behavior in hamsters. Physiol Behav.

[CR33] Flik G, Klaren PHM, Van den Burg EH, Metz JR, Huising MO (2006). CRF and stress in fish. Gen Comp Endocrinol.

[CR34] Fryer J, Lederis K, Rivier J (1985). ACTH-releasing activity of urotensin I and ovine CRF: interactions with arginine vasotocin, isotocin and arginine vasopressin. Regul Pept.

[CR35] Fulenwider HD, Caruso MA, Ryabinin AE (2021). Manifestations of domination: assessments of social dominance in rodents. Genes Brain Behav.

[CR36] Gammie SC, Hasen NS, Stevenson SA, Bale TL, D'Anna KL (2005). Elevated stress sensitivity in corticotropin-releasing factor receptor 2 deficient mice decreases maternal, but not intermale aggression. Behav Brain Res.

[CR37] Gillies GE, Linton EA, Lowry PJ (1982). Corticotropin releasing activity of the new CRF is potentiated several times by vasopressin. Nature.

[CR38] Gobrogge KL, Liu Y, Young LJ, Wang Z (2009). Anterior hypothalamic vasopressin regulates pair-bonding and drug-induced aggression in a monogamous rodent. Proc Natl Acad Sci.

[CR39] Godwin J, Thompson R (2012). Nonapeptides and social behavior in fishes. Horm Behav.

[CR40] Goodson JL (1998). Vasotocin and vasoactive intestinal polypeptide modulate aggression in a territorial songbird, the violet-eared waxbill (Estrildidae: *Uraeginthus granatina*). Gen Comp Endocrinol.

[CR41] Goodson JL, Adkins-Regan E (1999). Effect of intraseptal vasotocin and vasoactive intestinal polypeptide infusions on courtship song and aggression in the male zebra finch (*Taeniopygia guttata*). J Neuroendocrinol.

[CR42] Goodson JL, Bass AH (2001). Social behavior functions and related anatomical characteristics of vasotocin/vasopressin systems in vertebrates. Brain Res Rev.

[CR43] Goymann W, Wingfield JC (2004). Allostatic load, social status and stress hormones: the costs of social status matter. Anim Behav.

[CR44] Greenwood AK, Wark AR, Fernald RD, Hofmann HA (2008). Expression of arginine vasotocin in distinct preoptic regions is associated with dominant and subordinate behaviour in an African cichlid fish. Proc R Soc B.

[CR45] Harbuz MS, Chalmers J, De Souza L, Lightman SL (1993). Stress-induced activation of CRF and c-fos mRNAs in the paraventricular nucleus are not affected by serotonin depletion. Brain Res.

[CR46] Heinrichs SC, Koob GF (2004). Corticotropin-releasing factor in brain: a role in activation, arousal, and affect regulation. J Pharmacol Exp Ther.

[CR47] Huising M, Metz JR, van Schooten C, Taverne-Thiele A, Hermsen T, Verburg-van Kemenade B, Flik G (2004). Structural characterisation of a cyprinid (*Cyprinus carpio* L.) CRH, CRH-BP and CRH-R1, and the role of these proteins in the acute stress response. J Mol Endocrinol.

[CR48] Imaki T, Nahan J, Rivier C, Sawchenko P, Vale W (1991). Differential regulation of corticotropin-releasing factor mRNA in rat brain regions by glucocorticoids and stress. J Neurosci.

[CR49] Konno N, Kurosawa M, Kaiya H, Miyazato M, Matsuda K, Uchiyama M (2010). Molecular cloning and characterization of V2-type receptor in two ray-finned fish, gray bichir, *Polypterus senegalus* and medaka, *Oryzias latipes*. Peptides.

[CR50] Koob GF, Heinrichs SC (1999). A role for corticotropin releasing factor and urocortin in behavioral responses to stressors. Brain Res.

[CR51] Larson ET, O'Malley DM, Melloni J, Richard H (2006). Aggression and vasotocin are associated with dominant–subordinate relationships in zebrafish. Behav Brain Res.

[CR52] Lastein S, Höglund E, Øverli Ø, Døving KB (2008). Effects of antalarmin, a CRF receptor 1 antagonist, on fright reaction and endocrine stress response in crucian carp (*Carassius carassius*). J Comp Physiol A.

[CR53] Lema SC (2010). Identification of multiple vasotocin receptor cDNAs in teleost fish: sequences, phylogenetic analysis, sites of expression, and regulation in the hypothalamus and gill in response to hyperosmotic challenge. Mol Cell Endocrinol.

[CR54] Lema SC, Nevitt GA (2004). Exogenous vasotocin alters aggression during agonistic exchanges in male Amargosa River pupfish (*Cyprinodon nevadensis* amargosae). Horm Behav.

[CR55] Lema SC, Nevitt GA (2004). Variation in vasotocin immunoreactivity in the brain of recently isolated populations of a death valley pupfish, *Cyprinodon nevadensis*. Gen Comp Endocrinol.

[CR56] Lowry CA, Moore FL (2006). Regulation of behavioral responses by corticotropin-releasing factor. Gen Comp Endocrinol.

[CR57] Manning M, Stoev S, Chini B, Durroux T, Mouillac B, Guillon G (2008). Peptide and non-peptide agonists and antagonists for the vasopressin and oxytocin V1a, V1b, V2 and OT receptors: research tools and potential therapeutic agents. Prog Brain Res.

[CR58] McIntyre DC, Healy LM, Saari M (1979). Intraspecies aggression and monoamine levels in rainbow trout (*Salmo gairdneri*) fingerlings. Behav Neural Biol.

[CR59] Mele A, Cabib S, Oliverio A, Melchiorri P, Puglisi-Allegra S (1987). Effects of corticotropin releasing factor and sauvagine on social behavior of isolated mice. Peptides.

[CR60] R Core Team (2020). R: a language and environment for statistical computing.

[CR61] Risbrough VB, Stein MB (2006). Role of corticotropin releasing factor in anxiety disorders: a translational research perspective. Horm Behav.

[CR62] Robison CL, Meyerhoff JL, Saviolakis GA, Chen WK, Rice KC, Lumley LA (2004). A CRH1 antagonist into the amygdala of mice prevents defeat-induced defensive behavior. Ann NY Acad Sci.

[CR63] Rotzinger S, Lovejoy DA, Tan LA (2010). Behavioral effects of neuropeptides in rodent models of depression and anxiety. Peptides.

[CR64] RStudio Team (2019). RStudio: integrated development environment for R.

[CR65] Saito D, Komatsuda M, Urano A (2004). Functional organization of preoptic vasotocin and isotocin neurons in the brain of rainbow trout: central and neurohypophysial projections of single neurons. Neuroscience.

[CR66] Santangelo N, Bass A (2006). New insights into neuropeptide modulation of aggression: field studies of arginine vasotocin in a territorial tropical damselfish. Proc R Soc B.

[CR67] Semsar K, Kandel FLM, Godwin J (2001). Manipulations of the AVT system shift social status and related courtship and aggressive behavior in the bluehead wrasse. Horm Behav.

[CR68] Sloman KA, Armstrong JD (2002). Physiological effects of dominance hierarchies: laboratory artefacts or natural phenomena?. J Fish Biol.

[CR69] Smagin GN, Heinrichs SC, Dunn AJ (2001). The role of CRH in behavioral responses to stress. Peptides.

[CR70] Summers CH, Winberg S (2006). Interactions between the neural regulation of stress and aggression. J Exp Biol.

[CR71] The EFSA Journal (2009). Statement of EFSA prepared by the AHAW panel on: knowledge gaps and research needs for the welfare of farmed fish. EFSA J.

[CR72] Tonon MC (1986). Comparative effects of corticotropin-releasing factor, arginine vasopressin, and related neuropeptides on the secretion of ACTH and [alpha]-MSH by frog anterior pituitary cells and neurointermediate lobes in vitro. Gen Comp Endocrinol.

[CR73] Vale W, Spiess J, Rivier C, Rivier J (1981). Characterization of a 41-residue ovine hypothalamic peptide that stimulates secretion of corticotropin and beta-endorphin. Science.

[CR74] Veenema AH, Meijer OC, de Kloet ER, Koolhaas JM, Bohus BG (2003). Differences in basal and stress-induced HPA regulation of wild house mice selected for high and low aggression. Horm Behav.

[CR75] Wendelaar Bonga SE (1997). The stress response in fish. Physiol Rev.

[CR76] Winberg S, Nilsson GE (1993). Roles of brain monoamine neurotransmitters in agonistic behaviour and stress reactions, with particular reference to fish. Comp Biochem Physiol Part C.

[CR77] Young LJ, Winslow JT, Nilsen R, Insel TR (1997). Species differences in V1a receptor gene expression in monogamous and nonmonogamous voles: behavioral consequences. Behav Neurosci.

[CR78] Young LJ, Nilsen R, Waymire KG, MacGregor GR, Insel TR (1999). Increased affiliative response to vasopressin in mice expressing the V1a receptor from a monogamous vole. Nature.

